# Occupational Injuries of Spanish Wildland Firefighters: A Descriptive Analysis

**DOI:** 10.3390/healthcare12161615

**Published:** 2024-08-13

**Authors:** Fabio García-Heras, Juan Rodríguez-Medina, Arkaitz Castañeda, Patxi León-Guereño, Jorge Gutiérrez-Arroyo

**Affiliations:** 1 VALFIS Research Group, Department of Physical Education and Sports, Institute of Biomedicine (IBIOMED), University of León, 24071 León, Spain; jrodm@unileon.es; 2 Health, Physical Activity and Sports Science Laboratory, Department of Physical Activity and Sports, Faculty of Education and Sport, University of Deusto, 48007 Bilbao, Spain; arkaitz.castaneda@deusto.es (A.C.); patxi.leon@deusto.es (P.L.-G.)

**Keywords:** occupational injuries, wildland firefighters, physical employment, occupational health

## Abstract

The work of wildland firefighters, especially of the so-called ‘Brigadas de Refuerzo contra Incendios Forestales’, is characterised by high physical demands and extreme operating conditions. These professionals face long workdays (12 h), walking with heavy loads (~25 kg), being exposed to high temperatures (>30 °C), and handling specialised tools in high-risk environments. This study aimed to describe the prevalence of occupational injuries among members of the ‘Brigadas de Refuerzo contra Incendios Forestales’ and its relationship to variables such as age and work experience. A total of 217 wildland firefighters (18 female and 199 male) correctly answered a questionnaire developed on an ad hoc basis to meet the study’s objectives. A high prevalence of occupational injuries was observed among them (~76%). Age and work experience were shown to be significantly associated with injuries. Individuals over 35 years of age with more than 10 years’ experience had a higher probability of injury (OR = 2.14, CI = 1.12–4.06 and OR = 2.46, CI = 1.30–4.67, respectively). Injuries occurred mainly during physical training (~46%), followed by preventive work (~33%) and forest fires (~20%). The most common injuries were tendonitis and muscle pain (~44% and ~21% respectively), followed by sprains (~21%). The results underline the need for physical activity programmes adapted to help wildland firefighters, especially older and more experienced individuals. The identification of risk factors such as age and work experience can contribute to the prevention and management of occupational injuries among this group of highly specialised forestry workers. Specific preventative measures during training are required to mitigate the risk of injury among these crews, who play a crucial role in protecting the environment and public safety.

## 1. Introduction

The work performed by wildland firefighters (WFFs) is characterised by extended periods of effort (10 to 12 h work shifts, often on consecutive days) [1], long walks carrying very heavy loads (20–25 kg) [2], the handling of different scraping, cutting, and extinguishing tools, and high thermal stress due to high temperatures when working near fires [3]. The ‘Brigadas de Refuerzo contra Incendios Forestales’ (BRIFs) are highly specialised helitack units within the Spanish WFF workforce [4]. Their main mission is to provide support to the different autonomous regions in Spain both in dealing with simultaneous fires and in operations aimed at controlling large fires [5]. The BRIFs perform different tasks during their workdays, including emergency work, preventive tasks, and physical and practical training supervised by exercise professionals [6]. Similarly to the work of structural firefighters, police officers, and military personnel, the nature and physical demands of BRIF crews’ operations entail unavoidable exposure to risk, both in the short term (injury or accident) and in the long term (chronic pain, work disability, etc.) [7]. An injury is considered occupational if an event or exposure in the work environment caused or contributed to the injury or significantly aggravated an existing injury [8]. These injuries have been related to socio-demographic factors such as age, sex, years worked, and type of work performed [8,9,10,11,12].

The high likelihood of occupational injury within emergency workers has been described internationally [9,10,12,13,14,15], particularly among firefighters (FFs). It has been reported that a firefighter is injured in the line of duty every 8 min, either on base or in responding to an emergency [8]. A study conducted in 2016 on a sample of professional firefighters (14,349 male and 642 female) over a 12-month period established that ~12% sustained some form of injury, with significant differences between injured and non-injured personnel in relation to sex, age, smoking, and alcohol consumption [16]. Another study, this time longitudinal, found that between 2003 and 2012, 177 out of every 1000 Australian professional firefighters were injured in the line of duty, with the most common sites of injury being the knees, lower back, shoulders, and ankles [17]. Another study focused on 19,000 Korean structural firefighters and found that ~12% of them had been injured over a 12-month period; the subjects’ experience was shown to be particularly relevant, as the less experienced suffered a higher rate of injury than the more experienced [18]. In contrast, there is a global lack of knowledge of occupational injuries among forestry personnel. The only injury data reported to date concerned US wildland firefighters, with 1304 occupational injuries having been sustained between 2003 and 2007, slips or falls being the main cause (~28%), followed by tool use and heavy loads (22%) [9,10]. This determined that there were multiple relationships in the likelihood of injury, and that age, sex, experience, and work stress were key factors [19]. It has been established that an injury does not directly lead to taking sick leave, which may or may not be the case, but in all cases it causes the worker discomfort and/or pain, and affects their quality of life and work [20].

Therefore, we hypothesise that the incidence of injuries among BRIF wildland firefighters may vary according to the individual’s age and experience and be influenced by the types of tasks performed. Given that there are no published data on the main injuries sustained by Spanish wildland firefighters, the main objective of this study is to describe occupational injuries among the BRIF crews. 

## 2. Materials and Methods

### 2.1. Characteristics of the Study

This is a retrospective, observational cross-sectional study, measured through a self-reported online questionnaire. The questionnaire consisted of 5 parts: 1. Demographic characteristics and informed consent (personal and occupational descriptive questions); 2. Characteristics related to physical activity; 3. Psychological characteristics (data not included); 4. Occupational injuries; and 5. Chronic pain. Once the subjects participating in the study gave their informed consent, they completed the questionnaire in no more than 15 min.

### 2.2. Sample

The BRIFs are deployed on 10 bases spread across the mainland of the Iberian Peninsula and an additional one on an island (La Palma, Canary Islands). They have an operational force of 557 workers, divided into three categories, namely specialist wildland firefighters (*n* = 450), forepersons or intermediate command (*n* = 67), and crew leaders or senior command (*n* = 40); the study questionnaire was sent to 100% of the BRIF members by electronic means. Of these potential respondents, 217 subjects (~38%) completed the questionnaire (age: 36.4 ± 6.3 years; height: 176.4 ± 6.5 cm; body mass: 78.2 ± 11.6 kg; body mass index: 25.1 ± 2.9 kgꞏm^−2^; time as a firefighter: 10.1 ± 5.8 years), of which 18 were female (100% of the female workers) and 199 were male (~38% of the male workers).

### 2.3. Procedure

During the months of January and February 2021, several online meetings were held with BRIF wildland firefighters from the Iberian Peninsula, in order to objectively contextualise this study. The meetings were attended by members of each of the different operational positions (specialist wildland firefighters, forepersons or intermediate command, and crew leaders or senior command), and of the Association of BRIF Workers (known as ATBRIFs). The participants, who included females and males of different age groups, detailed the type of work carried out in each position. Once the information had been extracted, a self-reported questionnaire was developed on an ad hoc basis using the Google Forms application with the aim of studying the main injuries sustained by BRIF professionals (Supplementary Materials). The questionnaire was designed based on questionnaires previously used in the occupational and sports literature [8,21,22]. Additionally, it has been previously employed in studies related to chronic pain among Spanish wildland firefighters [23]; these studies have demonstrated the questionnaire’s effectiveness in capturing relevant data pertaining to occupational injuries and related health issues.

Data were collected during February and March 2021, and the questionnaire was disseminated using Google Forms via email lists. The participants received instructions on how to complete the questionnaire and were offered assistance with any questions that they might have. They were also provided with information about the purpose of the study, which was approved by the Ethics Committee of the University of León (Spain) (025-2020, 22 July 2020), in accordance with the latest version of the Declaration of Helsinki, Fortaleza, Brazil (2013). 

### 2.4. Description of the Study Variables

Once the data had been collected, the resulting database was reviewed to address possible inconsistencies, duplicate information, or questionable identifications. The results were analysed considering sex (male or female), age (≤35 years, >35 years), experience (≤10 and >10 years), and each subject’s job position (specialist, foreperson, or crew leader) [23]. In order to assess the positive effect of physical activity on injury prevention, the results were also analysed taking into account the number of hours spent performing physical activity per week, and whether the subjects performed any specific training to prevent injuries (yes/no) [24,25,26]. Physical activity was deemed to be any exercise or training performed by the wildland firefighters in their free time. Following previous recommendations established for healthy adults [27,28], physical activity was classified as low (1–3 h), moderate (3–5 h), and high (>5 h). 

Injuries were divided into 5 categories [8,9]: 1. Activity performed at the time of injury (physical and practical training, preventive work, forest fire, other activities). 2. Time of injury (previous year, or more than one year ago). 3. Cause of injury (impact or overuse); impact injuries were defined as those resulting from traumatic incidents such as accidents, falls, or slips. On the other hand, overuse injuries were categorided as those caused by repetitive actions, overloading, and similar factors. This classification allowed for the differentiation between acute, traumatic events and chronic conditions arising from repetitive strain. 4. Type of injury (tendonitis and muscle pain, sprains and osteoarticular pain, burns and blisters, muscle rupture, wounds and contusions, fractures, and others). 5. Location of injury: (i) lower limb (LL), which includes hip, knees, and ankles; (ii) upper limb (UL), which includes neck, shoulders, elbows, hands, and wrists; (iii) back, which includes lower back and thoracic spine; (iv) head; and (v) other. 

### 2.5. Statistical Analysis

All statistical analyses were performed using jamovi software version 2.3.38. Quantitative demographic data are presented as mean ± standard deviation, and the prevalence of categorical data responses is reported in terms of frequencies and percentages. A normality analysis was carried out using the Kolmogorov–Smirnov test, which assessed whether the quantitative data followed a normal distribution, something essential to ensure the validity of subsequent statistical tests. A Student’s *t*-test for independent samples was performed for the analysis between sexes and injured and non-injured. Effect size was calculated using Cohen’s d test, where values <0.20, 0.20–0.50, 0.51–0.80, and >0.80 were considered trivial, small, moderate, and large, respectively [29]. Frequencies and percentages were calculated to describe the distribution of relevant categorical variables, using the chi-square test to assess the significance of observed differences. For the variables for which the chi-square test was significant, the Odds Ratio (OR) and 95% confidence intervals (CIs) were applied to quantify the association between categorical variables and the precision of these estimates. The level of significance was set at *p* < 0.05. 

## 3. Results

A total of 217 BRIF members answered the questionnaire correctly. This represents ~38% of the total number of BRIF workers; 100% of the female participants answered the questionnaire (*n* = 18), while male respondents accounted for ~36% of the total male BRIF workers (*n* = 552). Table 1 shows the results regarding the sociodemographic variables of the sample. It shows that the female participants were ~5% smaller and ~17% lighter and had a lower BMI than the male participants (~9%).

Table 2 presents the results of the sociodemographic variables and the injuries sustained by the participants. Both the total sample and the male WFFs who reported injuries were significantly older than those who had not been injured (8.9% and 9.3%, respectively) and had more extensive work experience (34.3% and 36.1%, respectively). For female WFFs, no significant differences were found for any of the variables, but large effect sizes were observed for body mass index (BMI) and moderate for the weight of equipment used during their deployments (d = 0.970 and 0.570, respectively).

Of the 217 respondents, ~76% (*n* = 165) reported having sustained at least one occupational injury (Table 3), of whom 44 (2 female and 42 male WFFs) reported an injury in the previous year, representing ~20%, ~11%, and ~21% of the sample in total values, female, and male, respectively. No significant differences were observed between the injured and non-injured WFFs in terms of sex, weight of personal protective equipment, preventive training, job position, and weekly training hours. However, our results indicated a higher propensity for injury among participants aged 35 and above (OR = 2.14, CI = 1.12–4.06) and among those with more than 10 years’ experience (OR = 2.46, CI = 1.30–4.67). The job position variable was recoded into WFFs and leaders (foreperson and crew leader) for further analysis, with no significant differences found between these groups (OR = 1.60, CI = 0.743–3.460; χ^2^ = 1.47, *p* = 0.226).

A total of the 165 WFFs reported having sustained occupational injuries. Of these, 332 injuries were documented (28 suffered by female and 304 by male WFFs). Sixty-two of these injuries (~19%) had occurred in the last year, three to female WFFs and three hundred and four to male WFFs~ (11% and ~19% respectively). Significant differences between groups were observed for activity (χ^2^ = 11.9, *p*= 0.008), cause of injury (χ^2^ = 5.33, *p* = 0.021), and type of injury (χ^2^ = 12.4, *p* = 0.053) (Table 4). A 2 × 2 Odds Ratio analysis was performed on groups with differences (*p* ≤ 0.05) to establish significant relationships. Injuries occurred predominantly during physical training (45.8%), especially during continuous training or running (~75%), followed by preventive work (32.5%). Compared to leaders, WFFs showed a higher propensity to be injured during preventive work (OR = 0.195, CI = 0.0659–0.5790; χ^2^ = 10.3, *p* = 0.001). In addition, WFFs had a higher percentage of overuse injuries compared to leaders (OR = 2.22, CI = 1.11–4.42), while leaders reported a higher percentage of impact injuries (OR = 2.21, CI = 1.11–4.40). In terms of injury type, 44% were tendonitis and muscle pain, followed mainly by sprains and osteoarticular pain (~21%), with a similar distribution between both groups. In terms of location, half of the injuries were reported in the lower extremities, with a homogeneous distribution between WFFs and leaders (48.6% and 50.0%, respectively), followed by upper extremities and back (22.9% and 21.1%, respectively), with a similar distribution between the groups in all locations. 

## 4. Discussion

The main finding of our study was the high prevalence of occupational injuries reported by the WFFs in the BRIFs (~76%), and the relationship between these injuries and age and years’ experience. The prevalence analysed was higher than previously reported in other physically demanding professions such as structural firefighters (~66%) [30], police (66%) [31], and military (~34%) [32]. However, the prevalence of injuries in the previous year was lower than that reported by structural firefighters (~27%) [8].

Our results show that the participants over 35 years of age were twice as likely to suffer an occupational injury while carrying out their work (OR = 2.14, CI = 1.12–4.06). This phenomenon could be explained by the tendency of older individuals to be more frequently injured [9,10,33], and has already been documented in studies conducted in US WFFs, where a higher incidence of injury has been observed in those over 33 years of age [9,10]. In the case of structural firefighters, it has been noted that those over 42 years of age have a higher prevalence and severity of musculoskeletal disorders [34] and physical limitations that condition job performance in those over 45 years old [12,35], and that older structural firefighters require a longer recovery period [36]. It could be speculated that as the age of the WFFs in this study increased, their physical fitness may have decreased, which may have resulted in higher demands in performing their usual tasks and a higher risk of injury [37]. Furthermore, longer work experience (>10 years) doubled the likelihood of occupational injury among the WFFs in our study (OR = 2.46, CI = 1.30–4.67). The systematic repetition of movements involved in their various jobs (e.g., carrying heavy loads and handling hand tools) [38] could have caused overuse injuries, as has already been reported in structural firefighters with more than 15 years of service [34]. If the injury lasts three months or more, it is considered chronic pain [39], and this has been related to age (>35 years) and experience (>10 years) among WFFs, quadrupling and doubling the likelihood of this symptom occurring [23]. All this highlights the need to implement training programmes which are adapted and seek to prevent injuries in older and more experienced subjects [24,40,41]. These programmes could include ergonomic techniques to optimise postures and movements during work activities, as well as muscle strengthening exercises targeting vulnerable areas such as the back, shoulders, and knees. Additionally, training in safe lifting techniques and the proper use of hand tools could reduce the incidence of overuse injuries. Integrating educational sessions on chronic pain management and self-care strategies would also be relevant to support more experienced forest workers.

No differences have been observed in the literature in the frequency and percentage of injuries by sex, with similar results for physically active people [42,43]. However, previous research has noted that the incidence of injury among military personnel is higher for females than for males, with ~45 to ~57% of females in the military and ~27 to ~46% of males in the military having been injured [44]. This higher incidence of injury among military personnel could be explained by the absolute differences in the loads they must carry during their interventions. While the mean weight carried in our sample was 10.7 ± 3.7 kg, the absolute loads carried by military personnel during their interventions and training are much higher, with a mean weight of ~25 kg, and reaching a weight of up to ~45 kg [32]. In this regard, it is relevant to highlight the differences in height between injured and non-injured males in our study, with measurements of 178.7 ± 7.6 cm and 176.6 ± 5.6 cm, respectively. In this context, it is essential to highlight that differences in the load to be carried are not only limited to body size and sex, but also to specific regulations in different countries. In Spain, the standard weight of the fire extinguisher backpack used during WFF deployments is around 20–23 kg. This must be carried by all wildland firefighters [5]. This means that, regardless of sex or body size, all firefighters must carry the same load. However, given that females tend to have a smaller average height than men, it is plausible that females carry a higher percentage of their body weight compared to males [25,41]. This could predispose them to fatigue more quickly and increase their risk of sustaining musculoskeletal injuries, especially when considering the additional loads they must carry during interventions [25,45], as mentioned above. Therefore, when designing injury prevention strategies and assigning tasks within the forest fire brigades, important factors to be taken into account would be not only sex, but also body size.

Significant differences in the type of activity at the time of injury were observed (χ^2^= 11.9, *p*= 0.008). Injuries predominantly occurred during physical training (~46%), followed by preventive work (~33%) and firefighting operations (~20%). Similar patterns are observed among military personnel [46,47] and structural firefighters [48], where injuries also occur frequently during physical training aimed at enhancing cardiorespiratory fitness (~60% and ~33%, respectively). While improving cardiorespiratory endurance is recognised as potentially preventive, it is noted that participation in such activities (e.g., running) can also increase injury risks in physically demanding occupations [24]. The relationship between physical fitness and injury risk in occupational settings is intricate due to multiple contributing factors [49].

It was also found that the injuries sustained by the participating wildland firefighters occurred more frequently during preventive work, while leaders had a higher propensity to be injured during training (OR = 0.195, CI = 0.0659–0.5790; χ^2^ = 10.3, *p* = 0.001). This discrepancy suggests an intrinsic relationship between specific work activities and injury risk. Outside the high-risk wildfire season (from approximately November to May), in addition to regular training (2 days/week), wildland firefighters mainly perform preventive work such as the clearing and felling of tree trunks and branches, as well as the use of cutting and scraping tools, activities that demand high energy expenditure (from 5 to 17 METs) [50,51] and increase the risk of overuse injury [52]. In contrast, managers are exempted from preventive tasks, and spend their time mainly on coordination and management tasks, complementing these responsibilities with regular physical–practical training at the base. 

While other studies conducted with WFFs [9,10,19] and structural firefighters [8] have established impact as the main determinant of injury (falls, blows, or overexertion associated with specific one-off tasks), in our case the distribution was homogeneous across the board (~52% and ~48% in overuse and impact, respectively). In contrast, the WFFs in our study reported twice the risk of injury caused by repetitive tasks (OR = 2.22, CI = 1.11–4.42) and leaders for impact (OR = 2.21, CI = 1.11–4.40). This fact could again be determined by the tasks they have to perform, as WFFs use hand tools and perform repetitive movements for long hours, either in forest fires or in preventive work, which increases the likelihood of overuse injuries [33,53].

The typology of injuries was mainly tendonitis and muscle soreness, followed by sprains (~44 and ~21%, respectively). These results differ from other studies conducted with US WFFs, where sprains accounted for ~30% of the documented injuries [9,10]. This disparity could be determined by methodological differences while performing the work by both groups of WFFs. While Spanish WFFs mainly perform direct and indirect flame attack work, with working hours rarely exceeding 12 h during their interventions [3,38], US WFFs are mainly engaged in indirect attack with heavier loads, and often face interventions that last for several days, leading to more pronounced physical and mental fatigue [41,54,55]. This difference in work patterns could explain a higher incidence of one-off accidents such as falls and slips, which are the main factor of injury among US WFFs [9,10]. When comparing our data with those of structural firefighters, it can be seen that the injury typologies do not coincide [8,30,48]. While these studies on structural firefighters made a joint classification of tendonitis and muscle pain together with sprains and osteoarticular pain, following a recent expert consensus, we decided to separate these types of injuries in our study, because they involve different structures [56]. The overall percentage of injuries sustained by structural firefighters was around 80%, while in our study it was around ~65%. 

The location of the injuries can be seen in Table 4. They were mainly reported in the lower limbs (~49%), followed by the upper limbs and neck (~23%), and back (~21%). In general, the lower limbs are the most injured part in structural firefighters [8,12,48], police [32], and military personnel [53,57], determined by the type of work they perform, which mainly involves carrying heavy loads, with sporadic overexertion involving high activation of the lower body musculature. However, our results for injuries sustained to the lower limbs and back indicated that the incidence was higher among Spanish WFFs than among US WFFs (35 and 9%, respectively) [9,10], and lower in the case of the upper limbs and neck, where the US WFFs reported ~40% of injuries. Again, it is plausible that differences in the type of work to be performed and the loads to be carried could account for this disparity in results. 

The findings of this study highlight the need to implement regular physical activity programmes tailored to the specific demands of wildland firefighters. The high prevalence of occupational injuries, especially among individuals older than 35 and those with more than 10 years’ experience, underlines the importance of actively addressing the physical health of these professionals along with the influence of age, as noted in other studies [40,58]. Given the trend towards overuse injuries among the WFFs who perform repetitive manual labour, and impact injuries among those in leader roles during training, it is essential to design prevention strategies that address these different injury modalities. In addition, the fact that injuries were seen to affect different parts of the body, such as the lower limbs and back, suggests the need for specific approaches to strengthen and protect these vulnerable areas. When considering the diversity of tasks and responsibilities within the wildland fire service, it would be interesting to develop training programmes to meet the individual needs of each member, thus promoting the long-term health and well-being of these brave professionals. Furthermore, considering that these professionals often perform tasks at intensities exceeding 60% of their maximum oxygen consumption and handle heavy tools, it would be beneficial to incorporate training sessions that simulate these exertion levels [59]. This could involve high-intensity circuit training, which has proven effective in enhancing strength and work capacity among Spanish wildland firefighters [60]. Additionally, safe lifting techniques and stabilisation exercises could be introduced to strengthen vulnerable areas such as the back, shoulders, and knees, prone to injuries. This approach could optimise the physical readiness of wildland firefighters and mitigate the risk of injuries during their operational tasks. 

This study is not without limitations. Firstly, the data for self-selected participants and those who agreed to participate in the study may differ significantly from the data for those who chose not to participate, which could have introduced bias into the results. In addition, retrospective injury data collection may be subject to recall bias and lack of detailed documentation, which could affect the validity of the results. While there is some limitation between reported and actually certified injuries, it has been noted that a high percentage of self-reported injuries were accurate compared to medical records among the military population [47], which supports the use of survey data for the assessment of injury outcomes. Furthermore, the questionnaire utilised in this study was the same one used in studies related to wildland firefighters [23] and was developed based on specific literature in the field of occupational [8] and sports injuries [21], which supports its relevance and applicability. However, it is important to note that this questionnaire has not undergone a formal validation process. This lack of validation could be considered a limitation, and future studies should aim to formally validate the questionnaire to ensure its robustness. Finally, it should be noted that despite not having surveyed the entire BRIF wildland firefighter population, the sample obtained represented approximately 38.75% of the total population and provided us with a solid basis for drawing inferences about the population as a whole. The high response rate, together with the 100% participation of female BRIF members in our sample, contributed to strengthening the validity of our data.

## 5. Conclusions

This study is the first comprehensive analysis of occupational injuries among Spanish wildland firefighters. Our findings show that ~76% of the surveyed WFFs (specifically, BRIFs) reported having sustained at least one occupational injury, with a total of 332 documented injuries. Among the most notable findings, a higher propensity for injury was observed among participants over 35 years of age and those with more than 10 years’ experience. Significant differences between the groups in terms of activity, cause of injury, and type of injury were also evident, with WFFs showing a higher propensity to be injured during preventive and overuse work compared to managers. These results provide valuable information to improve the prevention and management of occupational injuries among the members of these important crews.

## Figures and Tables

**Table 1:** Sociodemographic variables.

	Total (217)	Female (18)	Male (199)	*p*	d
Age (years)	36.3 ± 6.3	36.6 ± 8.8	36.2 ± 6.1	0.836	0.052
Mass (kg)	78.2 ± 11.6	66.1 ± 7.2	79.4 ± 11.3	<0.001	1.403
BMI (kg·m^−2^)	25.1 ± 2.9	23.1 ± 1.3	25.3 ± 2.9	0.002	0.978
Height (cm)	176.4 ± 6.5	169.2 ± 6.4	177.1 ± 6.2	<0.001	1.253
Experience (years)	10.1 ± 5.8	8.9 ± 5.4	10.2 ± 5.8	0.385	0.231
PE mass (kg)	11.8 ± 5.5	10.7 ± 3.7	12.0 ± 5.6	0.34	0.273

*p*, significant differences between female and male; d, effect size (indicates the magnitude of differences); BMI, body mass index; PE, personal equipment.

**Table 2:** Injured/non-injured results by sex.

	Total (217)			Female WFFs (18)		Male WFFs (199)			
	Injured (165)	Not Injured (52)	d	Injured (13)	Not Injured (5)	d	Injured (152)	Not Injured (47)	d
Age (years)	37.1 ± 6.1 *	33.8 ± 6.4	0.528	37.1 ± 8.3	35.2 ± 10.7	0.209	37.1 ± 5.9 *	33.6 ± 6.0	0.581
Mass (kg)	78.4 ± 11.3	77.7 ± 12.6	0.055	66.9 ± 8.1	63.8 ± 3.8	0.430	79.4 ± 11.0	79.2 ± 12.3	0.016
BMI (kg·m^−2^)	25.3 ± 2.9	24.5 ± 2.9	0.275	23.4 ± 1.2	22.2 ± 1.3	0.970	25.5 ± 2.9	24.7 ± 3.0	0.248
Height (cm)	176.0 ± 6.1	177.8 ± 7.8	0.230	169.0 ± 7.3	169.6 ± 3.3	0.092	176.6 ± 5.6 *	178.7 ± 7.6	0.335
Experience (years)	11.0 ± 5.7 *	7.2 ± 5.2	0.731	9.2 ± 5.5	8.2 ± 5.5	0.186	11.1 ± 5.7 *	7.1 ± 5.2	0.725
PE mass (kg)	11.9 ± 5.7	11.9 ± 4.8	0.000	10.1 ± 3.0	12.2 ± 5.4	0.570	12.0 ± 5.9	11.8 ± 4.8	0.034

*, significant differences between injured and non-injured participants (*p* ≤ 0.05); d, effect size; WFFs, wildland firefighters; BMI, body mass index; PE, personal equipment.

**Table 3:** Proportions of injured/non-injured.

Grouping		Injured	Not Injured	Total	χ^2^ (*p*)
Total		165 (76.0%)	52 (24%)	217 (100.0%)	
Sex	female	13 (72.2%)	5 (27.8%)	18 (100%)	0.157 (0.692)
	male	152 (76.4%)	47 (23.6%)	199 (100%)	
Age	>35 years	91 (82.7%)	19 (17.3%)	110(100.0%)	5.48 (0.019)
	≤35 years	74 (69.2%)	33 (30.8%)	107 (100.0%)	
Position	WFF	139 (77.7%)	40 (22.3%)	179 (100.0%)	1.99 (0.369)
	foreperson	20 (71.4%)	8 (28.6%)	28 (100.0%)	
	Crew leader	6 (60.0%)	4 (40.0%)	10 (100.0%)	
Experience	>10 years	100 (83.3%) *	20 (16.7%)	120 (100.0%)	7.84 (0.005)
	<10 years	65 (67.0%)	32 (33.0%)	97 (100.0%)	
Physical activity (hours)	<3 h	38 (79.2%)	10 (20.8%)	48 (100.0%)	0.333 (0.847)
	3–5 h	54 (75.0%)	18 (25.0%)	72 (100.0%)	
	>5 h	73 (75.3%)	24 (24.7%)	97 (100.0%)	
PE (kg)	>10 kg	113 (73.4%)	41 (26.6%)	154 (100.0%)	2.06 (0.151)
	<10 kg	52 (82.5%)	11 (17.5%)	63 (100.0%)	
Preventive training	Yes	122 (78.2%)	34 (21.8%)	156 (100.0%)	1.43 (0.231)
	No	43 (70.5%)	18 (29.5%)	61 (100.0%)	

*, significant differences within years’ experience groups >10 years and <10 years (*p* ≤ 0.05); χ^2^, chi-square analysis; *p*, significance level; WFFs, wildland firefighters; PE, personal equipment.

**Table 4:** Proportion of injuries according to type of activity, cause of injury, type of injury, and location of injury.

Grouping		Position			
		WFF	Leader	Total	χ^2^ (*p*)
Total		292 (88.0%)	40 (12.0%)	332 (100.0%)	-
Activity	Physical training	127 (43.5%)	25 (62.5%)	152 (45.8%)	11.9 (0.008)
	Preventive work	104 (35.6%) *	4 (10.0%)	108 (32.5%)	
	Wildland fire	56 (19.2%)	9 (22.5%)	65 (19.6%)	
	Others	5 (1.7%)	2 (5.0%)	7 (2.1%)	
Cause of injury	Overuse	159 (54.5%) *	14 (35.0%)	173 (52.1%)	5.33 (0.021)
	Impact	133 (45.5%)	26 (65.0%) *	159 (47.9%)	
Type of injury	Tendonitis and muscle pain	133 (45.5%)	13 (32.5%)	146 (44.0%)	12.4 (0.053)
	Sprains and osteoarticular pain	64 (21.9%)	7 (17.5%)	71 (21.4%)	
	Burns (blisters)	29 (9.9%)	2 (5.0%)	31 (9.3%)	
	Muscle–ligament break	26 (8.9%)	5 (12.5%)	31 (9.3%)	
	Wounds and bruises	15 (5.1%)	4 (10.0%)	19 (5.7%)	
	Fractures	15 (5.1%)	4 (10.0%)	19 (5.7%)	
	Other	10 (3.4%)	5 (12.5%)	15 (4.5%)	
Location	Lower limbs	142 (48.6%)	20 (50%)	162 (48.8%)	1.56 (0.815)
	Upper limbs	67 (22.9%)	9 (22.5%)	76 (22.9%)	
	Back	63 (21.6%)	7 (17.5%)	70 (21.1%)	
	Head	14 (4.8%)	2 (5.0%)	16 (4.8%)	
	Other	6 (2.1%)	2 (5.0%)	8 (2.4%)	

*, significant differences between WFFs and leaders (*p* ≤ 0.05); χ^2^, chi-square analysis; *p*, significance level; WFFs, wildland firefighters.

## Data Availability

The data presented in this study are available on request from the corresponding author. The data are not publicly available due to privacy restrictions.

## Supplementary Materials

The following supporting information can be downloaded at: https://www.mdpi.com/article/10.3390/healthcare12161615/s1, Questionnaire (Spanish and English version).
