# Supplementary material for: Occupational Injuries of Spanish Wildland Firefighters: A Descriptive Analysis
Source: Healthcare (Basel). 2024 Aug 13;12(16):1615. doi: 10.3390/healthcare12161615 (PMC11354085; doi:10.3390/healthcare12161615)
Supplement: Supplementary file 1 [file healthcare-12-01615-s001.zip › Questionnaire (English).pdf]

DESCRIPTIVE ANALYSIS OF THE MAIN INJURIES OF THE FOREST FIRE DEPARTMENTS (BRIF)

In general terms, the work of extinguishing forest fires is seen as an eminently physical task. very demanding, where the long duration of the effort, hostile and changing weather conditions, and the long duration of the effort lead the worker to a very high physical load. In other physical professions, such as structural and military firefighters, the main injuries and the potential risk of suffering them have been described in numerous studies, but this is not the case of forest firefighters. Therefore, the present questionnaire, created jointly by members of the University of Deusto-Donosti (patxi.leon@deusto.es), and the University of León (fgarh@unileon.es), and with the support of the Association of BRIF Workers (AT-BRIF), has the objective of knowing and being able to deepen in the typology of the injuries of the BRIF Forest Firefighters.

The questionnaire consists of 5 parts:
Part 1: sociodemographic variables (personal and occupational)
Part 2: physical activity performed in their daily lives
Part 3: behavioral and psychological aspects Part
4: injuries in the workplace Part5: chronic pain

Participation is completely voluntary and anonymous.

The processing, communication and transfer of personal data of all participating subjects will comply with the provisions of the Organic Law 3/2018 of 5 December on the Protection of Personal Data and guarantee of digital rights. In accordance with the provisions of the aforementioned legislation, you may exercise the rights of opposition and cancellation of data, for which you should contact the directors of the study. The researchers of the project may have access to the volunteer's data. The personal data and information obtained from this study, with guaranteed privacy for your identity, will be known only by the researchers of the project. By accepting this consent you authorize the collection, storage and analysis of your requested data, unlinked to your identity by a double reversible coding system. Those who continue the process will show their acceptance to the characteristics of the research and will ensure their understanding.

*Mandatory

1. 1.- I have read and accept the conditions: *

Mark only one oval.

- Yes
- No

SOCIO-DEMOGRAPHIC VARIABLES

In this section we ask questions about the personal and work characteristics of the participants, in order to contextualize the sample in an objective way.

2. 2.- Age: *

3. 3.- Sex: *

Mark only one oval.

- Woman
- Man
- Other

4. Weight in Kg: *

5. 5.- Height in cm: *

6. 6.- Are you a smoker? *

Mark only one oval.

- No
- Yes

7. 6.1- If you are a smoker, how many cigarettes do you smoke on a normal day?

Mark only one oval.

- 3 or less
- From 4 to 10
- From 11 to 15
- 16 or more

8. 7.- Do you consume alcohol? *

Mark only one oval.

- No
- Yes, occasionally (weekend)
- Yes, on a regular basis (daily)

9. 8.- Have you passed the COVID- 2019? *

Mark only one oval.

- No
- Don't know for sure
- Yes, asymptomatic
- Yes, mild symptoms
- Yes, severe symptoms without hospitalization
- Yes, severe symptoms with hospitalization

10. 8.1.- In the case of having passed the COVID- 19 disease, indicate when:

Mark only one oval.

- March 2020
- April 2020
- May 2020
- June 2020
- July 2020
- August 2020
- September 2020
- October 2020
- November 2020
- December 2020
- January 2021
- February 2021

11. 9.- Years of experience within the BRIF device: *

12. 10.- BRIF in which you are currently working (in the case of being assigned this year to Ruente, indicate the one in which you have your usual destination + the Ruente tab): *

Select all that apply.

- [ ] Tineo
- [ ] Laza
- [ ] Lubia
- [ ] Puerto del Pico
- [ ] Tabuyo del monte
- [ ] Daroca
- [ ] Pinofranqueado
- [ ] La Iglesuela
- [ ] Prado de los Esquiladores
- [ ] Puntagorda
- [ ] Ruente

13. 11.- Current position: *

Mark only one oval.

- ( ) Forestry firefighter specialist
- ( ) Forestry firefighter foreman
- ( ) Forestry firefighter technician

14. 12.- Approximate weight of PPE + accessories used (in kg) (e.g., water bottle, supplies, change of clothes, etc.):

____

15. 13.- Main tools used during the extinguishing campaign: (indicate the 2 most used ones)

Select all that apply.

- [ ] Fire extinguisher backpack
- [ ] Batefuegos
- [ ] Fire extinguisher backpack + fire bat
- [ ] Pulaski, hoe, gorgui, mcleod or similar
- [ ] Calabozo, tajamatas or similar
- [ ] Chainsaw, chainsaw loads, etc.
- [ ] Rotating tool in quadrant function
- [ ] Loading of material, food, supplies, etc.
- [ ] Management, control and/or organization tasks
- [ ] Other: ______________________________

16. 14.- Main tools/tasks performed during the preventive work phase (winter): *

Select all that apply.

- [ ] Brushcutter
- [ ] Chainsaw
- [ ] Other mechanical tools
- [ ] Management, organization and/or control tasks
- [ ] Hauling of branches or materials
- [ ] Other: ____________

17. 15.- In the case of participation in prescribed burns, and/or EPRIF, indicate the main tools/actions used/carried out:

Select all that apply.

- [ ] Drip torch
- [ ] Backpack fire extinguisher
- [ ] Fire extinguisher
- [ ] Brushcutter
- [ ] Chainsaw
- [ ] Hauling of branches or material
- [ ] Rotation of functions/tools according to quadrant
- [ ] Management, control and/or organization tasks
- [ ] Other: ____________

PHYSICAL ACTIVITY

In this section you will be asked questions about your daily physical activity.

18. 16.- How many hours on average do you train per week? *

Mark only one oval.

- ( ) <1
- ( ) 1-3
- ( ) 3-5
- ( ) 5-7
- ( ) >7

19. 17.- Normally, in a typical week: how many days a week are you physically active for at least 60 minutes? (Physical activity is any activity that increases your heart rate and makes you short of breath at some point. This physical activity can be done in any sport, playing games with friends, or walking to work) * * Physical activity is any activity that increases your heart rate and makes you short of breath at some point.

Mark only one oval.

No day
One day
Two days
Three
days Fou r
d a y s
F i v e d a y s
S i x
d a y s Six days
Seven
days

20. 18. -What type of physical activity do you do? *

Select all that apply.

Hiking
Hiking
Running
Strength training
Team sports (e.g. rugby, soccer, etc.)
Individual sports (e.g. tennis, canoeing, etc.)
Other:

21. 18.1- Do you perform specific exercises to strengthen the CORE? *

Mark only one oval.

No
Yes

22. 19.- Do you think you are an active person compared to other people your age? *

Mark only one oval.

Very
inactive
Inactive
Neutral
Active
Very

active

23. 20.- How would you describe your general health? *

Mark only one oval.

Very weak
Weak
Neutral
Good
Very good

24. 20.1- On a regular basis, the quality of your sleep was: *

Mark only one oval.

Very bad
Bad
Passable
Good
Very good

3RD PART: ASPECTS RELATED TO THE EMOTIONAL STATE

In the following section, 7 questions related to stress and moods are developed.

25. 21.- In the last month... How often have you felt unable to control important things in your life? *

Mark only one oval.

Never
Almost never
Occasionally
Frequently
Very often

26. 22.- In the last month... How often have you been confident in your ability to handle your problems? *

Mark only one oval.

Never
Almost never
Occasionally
Frequently
Very often

27. 23.- In the last month...How often have you felt that things were going well for you? *

Mark only one oval.

- Never
- Almost never
- Occasionally
- Frequently
- Very often

28. 24.- In the last month... How often have you felt that difficulties accumulated so much that you could not overcome them? *

Mark only one oval.

- Never
- Almost never
- Occasionally
- Frequently
- Very often

29. 25. Your thoughts are (where calm=0, and worries=10): *

Mark only one oval.

	0	1	2	3	4	5	6	7	8	9	10	
Calm												Concern

30. 26.- Your body feels (where relaxed= 0, and tense=10): *

Mark only one oval.

	0	1	2	3	4	5	6	7	8	9	10	
Relaxed												Tense

31. 27.- How do you feel (where safe= 0, and scared=10): *

Mark only one oval.

	0	1	2	3	4	5	6	7	8	9	10	
Insurance												Scared

PART 4: OCCUPATIONAL INJURIES

First and foremost, as described by Phelps et al, (2018), "An injury is considered work-related (occupational) if an event or exposure in the work environment caused or contributed to the injury or significantly aggravated an existing injury". Likewise, it is understood that the injury does not directly imply sick leave, but may or may not exist, but in all cases it produces discomfort and/or pain in the worker, conditioning the worker's quality of life and work (Scherzer et al, 2005).

Within the work environment we understand: fires or emergencies (displacement, action or return), basic physical training, technical-tactical practices (marching,handling of tools, etc.), preventive work, prescribed burns, etc.

In this section you will find different questions related to work-related injuries.

32. 28.- Have you ever had a work-related injury? *

Mark only one oval.

- ◯ *No* *Skip to question 104*
- ◯ Yes

INJURY 1

33. 29.- If you have suffered a work-related injury, when did the injury occur? *

Mark only one oval.

- ◯ During the last year (between February 2020 and the present)
- ◯ More than one year ago

34. 30.- If the injury occurred within the last year, indicate the month in which it occurred:

Mark only one oval.

- ◯ February 2020
- ◯ March 2020
- ◯ April 2020
- ◯ May 2020
- ◯ June 2020
- ◯ July 2020
- ◯ August 2020
- ◯ September 2020
- ◯ October 2020
- ◯ November 2020
- ◯ December 2020
- ◯ January 2021
- ◯ February 2021

35. 31.- Job position you held at the time of the injury: *

Mark only one oval.

- Forestry Firefighter Specialist
- Forestry Firefighter
- Foreman
- Forestry Firefighter Technician

36. 32.- What type of injury was it? *

Mark only one oval.

- New injury
- Relapse

37. 33.-Activity you were performing when the injury occurred: *

Mark only one oval.

- Forest fire
- Other emergencies
- Base practices
- Physical training on the basis of
- Preventive work
- Other: ______________________

38. 33.1.- In the case of having suffered the injury during a "Forest Fire", indicate the work carried out at the time of the injury:

Mark only one oval.

- Approach
- Direct Attack
- Indirect Attack
- Mixed
- Surveillance
- Perimetration work, liquidation
- Withdrawal
- Not remembered
- Other: ______________________

39. 33.2.-In the case of having suffered the injury during "base practice", indicate the work performed at the time of the injury:

Mark only one oval.

Handling of tools (line of defense, practice of direct attack, etc.)
Marches with EPI
Gear with PPE + tool
Adequacy of material (cleaning, sharpening, etc.)
Shipments and landings
Not remembered
Other:

40. 33.3.-In the case of having suffered the injury during "basic physical training", indicate the type of training performed at the time of the injury:

Mark only one oval.

Strength training
Resistance training
Flexibility/mobility training
Not Recalled
Other:

41. 33.4.-In the case of having suffered the injury during "preventive work", indicate the type of task or work performed at the time of the injury:

Mark only one oval.

Clearing
Prescribed burns
Access
Freight transportation
Carrying of material and branches
Burning of material remains
Not recalled
Other:

42. 34.- Main cause of injury: *

Mark only one oval.

- Impact
- Overuse
- Unknown
- Other: ____________

43. 34.1.- In the case of having selected the option IMPACT, indicate the option that caused the injury:

Mark only one oval.

- Fall
- Jump
- Slip
- Stumble
- Hit by an object
- Other: ____________

44. 34.2.- In the case of having selected the OVERUSE option, indicate the option that caused the injury:

Mark only one oval.

- Excessive effort
- Repetitive tasks
- Exposure to flames
- Extreme weather (hot or cold)
- Other: ____________

45. 35.- Type of injury: *

Mark only one oval.

- Burn
- Heatstroke
- Inhalation of fumes
- Muscle pain
- Tendinitis
- Muscle rupture
- Osteo-articular sprain
- Joint dislocation
- Bone fracture
- Fracture
- Wound/cut, with abundant bleeding
- Concussion
- Stroke
- Blisters
- Other: ____________________

46. 36.- Injured body part: *

Mark only one oval.

- Head
- Neck
- Nose
- Eyes
- Ears
- Fingers-hand
- Hand
- Wrist
- Forearm
- Elbow
- Shoulder
- Collar bone
- Upper back (cervical area)
- Middle back (thoracic area)
- Lower back (lumbar area)
- Ribs
- Chest
- Abdomen
- Hip
- Pelvis
- Thigh
- Knee
- Leg
- Ankle
- Foot
- Toenails
- Toes
- Plantar fascia
- Heel
- Other: ____________________

47. 37.-Duration of the lesion: *

Mark only one oval.

- < 1 week
- 1-3 weeks
- 3-6 weeks
- > 6 weeks

48. 38.- The injury required medical attention: *

Mark only one oval.

No

Yes

49. 39.- Did the injury require sick leave or absence from work? *

Mark only one oval.

No

Yes

50. Have you had any other injuries on the job?

Mark only one oval.

NoSkip to question 104

Yes

INJURY 2

51. 29.- If you have suffered a work-related injury, when did the injury occur? *

Mark only one oval.

During the last year (between February 2020 and the present)

More than one year ago

52. 30.- If the injury occurred within the last year, indicate the month in which it occurred:

Mark only one oval.

- February 2020
- March 2020
- April 2020
- May 2020
- June 2020
- July 2020
- August 2020
- September 2020
- October 2020
- November 2020
- December 2020
- January 2021
- February 2021

53. 31.- Job position you held at the time of the injury: *

Mark only one oval.

- Forestry Firefighter Specialist
- Forestry Firefighter
- Foreman
- Forestry Firefighter Technician

54. 32.- What type of injury was it? *

Mark only one oval.

- New injury
- Relapse

55. 33.-Activity you were performing when the injury occurred: *

Mark only one oval.

- Forest Fire
- Other emergencies
- Base Practices
- Physical training on the basis
- Preventive work
- Other: ______________________

56. 33.1.- In the case of having suffered the injury during a "Forest Fire", indicate the work carried out at the time of the injury:

Mark only one oval.

- Approach
- Direct Attack
- Indirect Attack
- Mixed
- Surveillance
- Perimetration work, liquidation
- Withdrawal
- Not remembered
- Other: ____________

57. 33.2.-In the case of having suffered the injury during "base practice", indicate the work performed at the time of the injury:

Mark only one oval.

- Handling of tools (line of defense, practice of direct attack, etc.)
- Marches with EPI
- Gear with PPE + tool
- Adequacy of material (cleaning, sharpening, etc.)
- Shipments and landings
- Not remembered
- Other: ____________

58. 33.3.-In the case of having suffered the injury during "basic physical training", indicate the type of training performed at the time of the injury:

Mark only one oval.

- Strength training
- Resistance training
- Flexibility/mobility training
- Not Recalled
- Other: ____________

59. 33.4.-In the case of having suffered the injury during "preventive work", indicate the type of task or work performed at the time of the injury:

Mark only one oval.

- Clearing
- Prescribed burns
- Access
- Freight transportation
- Carrying of material and branches
- Burning of material remains
- Not recalled
- Other:

60. 34.- Main cause of injury: *

Mark only one oval.

- Impact
- Overuse
- Unknown
- Other:

61. 34.1.- In the case of having selected the option IMPACT, indicate the option that caused the injury:

Mark only one oval.

- Fall
- Jump
- Slip
- Stumble
- Hit by an object
- Other:

62. 34.2.- In the case of having selected the OVERUSE option, indicate the option that caused the injury:

Mark only one oval.

- Excessive effort
- Repetitive tasks
- Exposure to flames
- Extreme weather (hot or cold)
- Other:

63. 35.- Type of injury: *

Mark only one oval.

- Burn
- Heatstroke
- Inhalation of fumes
- Muscle pain
- Tendinitis
- Muscle rupture
- Osteo-articular sprain
- Joint dislocation
- Bone fracture
- Fracture
- Wound/cut, with abundant bleeding
- Concussion
- Stroke
- Blisters
- Other: ____________

64. 36.- Injured body part: *

Mark only one oval.

- Head
- Neck
- Nose
- Eyes
- Ears
- Fingers-hand
- Hand
- Wrist
- Forearm
- Elbow
- Shoulder
- Collar bone
- Upper back (cervical area)
- Middle back (thoracic area)
- Lower back (lumbar area)
- Ribs
- Chest
- Abdomen
- Hip
- Pelvis
- Thigh
- Knee
- Leg
- Ankle
- Foot
- Toenails
- Toes
- Plantar fascia
- Heel
- Other: ____________

65. 37.-Duration of the lesion: *

Mark only one oval.

- < 1 week
- 1-3 weeks
- 3-6 weeks
- > 6 weeks

66. 38.- Did the injury require medical attention?

Mark only one oval.

- No
- Yes

67. 39.- Did the injury require sick leave or absence from work? *

Mark only one oval.

- No
- Yes

68. Have you had any other injuries on the job? *

Mark only one oval.

- *No* *Skip to question 104*
- Yes

INJURY 3

69. 29.- If you have suffered a work-related injury, when did the injury occur? *

Mark only one oval.

- During the last year (between February 2020 and the present)
- More than one year ago

70. 30.- If the injury occurred within the last year, indicate the month in which it occurred:

Mark only one oval.

- February 2020
- March 2020
- April 2020
- May 2020
- June 2020
- July 2020
- August 2020
- September 2020
- October 2020
- November 2020
- December 2020
- January 2021
- February 2021

71. 31.- Job position you held at the time of the injury: *

Mark only one oval.

- Forestry Firefighter Specialist
- Forestry Firefighter
- Foreman
- Forestry Firefighter Technician

72. 32.- What type of injury was it? *

Mark only one oval.

- New injury
- Relapse

73. 33.-Activity you were performing when the injury occurred: *

Mark only one oval.

- Forest fire
- Other emergencies
- Base practices
- Physical training on the basis of
- Preventive work
- Other: ____________________

74. 33.1.- In the case of having suffered the injury during a "Forest Fire", indicate the work carried out at the time of the injury:

Mark only one oval.

Approach

Direct Attack

Indirect Attack

Mixed

Surveillance

Perimetration work, liquidation

Withdrawal

Not remembered

Other:

75. 33.2.-In the case of having suffered the injury during "base practice", indicate the work performed at the time of the injury:

Mark only one oval.

Handling of tools (line of defense, practice of direct attack, etc.)

Marches with EPI

Gear with PPE + tool

Adequacy of material (cleaning, sharpening, etc.)

Shipments and landings

Not remembered

Other:

76. 33.3.-In the case of having suffered the injury during "basic physical training", indicate the type of training performed at the time of the injury:

Mark only one oval.

Strength training

Resistance training

Flexibility/mobility training

Not Recalled

Other:

77. 33.4.-In the case of having suffered the injury during "preventive work", indicate the type of task or work performed at the time of the injury:

Mark only one oval.

- Clearing
- Prescribed burns
- Access
- Freight transportation
- Carrying of material and branches
- Burning of material remains
- Not recalled
- Other: __________

78. 34.- Main cause of injury: *

Mark only one oval.

- Impact
- Overuse
- Unknown
- Other: __________

79. 34.1.- In the case of having selected the option IMPACT, indicate the option that caused the injury:

Mark only one oval.

- Fall
- Jump
- Slip
- Stumble
- Hit by an object
- Other: __________

80. 34.2.- In the case of having selected the OVERUSE option, indicate the option that caused the injury:

Mark only one oval.

- Excessive effort
- Repetitive tasks
- Exposure to flames
- Extreme weather (hot or cold)
- Other: __________

81. 35.- Type of injury:

Mark only one oval.

- Burn
- Heatstroke
- Inhalation of fumes
- Muscle pain
- Tendinitis
- Muscle rupture
- Osteo-articular sprain
- Joint dislocation
- Bone fracture
- Fracture
- Wound/cut, with abundant bleeding
- Concussion
- Stroke
- Blisters
- Other: ____________________

82. 36.- Injured body part: *

Mark only one oval.

- Head
- Neck
- Nose
- Eyes
- Ears
- Fingers-hand
- Hand
- Wrist
- Forearm
- Elbow
- Shoulder
- Collar bone
- Upper back (cervical area)
- Middle back (thoracic area)
- Lower back (lumbar area)
- Ribs
- Chest
- Abdomen
- Hip
- Pelvis
- Thigh
- Knee
- Leg
- Ankle
- Foot
- Toenails
- Toes
- Plantar fascia
- Heel
- Other: ____________________

83. 37.-Duration of the lesion: *

Mark only one oval.

- < 1 week
- 1-3 weeks
- 3-6 weeks
- > 6 weeks

84. 38.- The injury required medical attention

Mark only one oval.

- No
- Yes

85. 39.- Did the injury require sick leave or absence from work? *

Mark only one oval.

- No
- Yes

86. Have you had any other injuries on the job? *

Mark only one oval.

- *NoSkip to question 104*
- Yes

INJURY 4

87. 29.- If you have suffered a work-related injury, when did the injury occur? *

Mark only one oval.

- During the last year (between February 2020 and the present)
- More than one year ago

88. 30.- If the injury occurred within the last year, indicate the month in which it occurred:

Mark only one oval.

- February 2020
- March 2020
- April 2020
- May 2020
- June 2020
- July 2020
- August 2020
- September 2020
- October 2020
- November 2020
- December 2020
- January 2021
- February 2021

89. 31.- Job position you held at the time of the injury: *

Mark only one oval.

- Forestry Firefighter Specialist
- Forestry Firefighter

Foreman Forestry Firefighter

Technician

90. 32.- What type of injury was it? *

Mark only one oval.

- New injury
- Relapse

91. 33.-Activity you were performing when the injury occurred: *

Mark only one oval.

- Forest fire

Other emergencies

Base practices

- Physical training on the basis

of Preventive work

- Other: ____________________

92. 33.1.- In the case of having suffered the injury during a "Forest Fire", indicate the work carried out at the time of the injury:

Mark only one oval.

- Approach
- Direct Attack
- Indirect Attack
- Mixed
- Surveillance
- Perimetration work, liquidation
- Withdrawal
- Not remembered
- Other: __________

93. 33.2.-In the case of having suffered the injury during "base practice", indicate the work performed at the time of the injury:

Mark only one oval.

- Handling of tools (line of defense, practice of direct attack, etc.)
- Marches with EPI
- Gear with PPE + tool
- Adequacy of material (cleaning, sharpening, etc.)
- Shipments and landings
- Not remembered
- Other: __________

94. 33.3.-In the case of having suffered the injury during "basic physical training", indicate the type of training performed at the time of the injury:

Mark only one oval.

- Strength training
- Resistance training
- Flexibility/mobility training
- Not Recalled
- Other: __________

95. 33.4.-In the case of having suffered the injury during "preventive work", indicate the type of task or work performed at the time of the injury:

Mark only one oval.

- Clearing
- Prescribed burns
- Access
- Freight transportation
- Carrying of material and branches
- Burning of material remains
- Not recalled
- Other: ______

96. 34.- Main cause of injury: *

Mark only one oval.

- Impact
- Overuse
- Unknown
- Other: ______

97. 34.1.- In the case of having selected the option IMPACT, indicate the option that caused the injury:

Mark only one oval.

- Fall
- Jump
- Slip
- Stumble
- Hit by an object
- Other: ______

98. 34.2.- In the case of having selected the OVERUSE option, indicate the option that caused the injury:

Mark only one oval.

- Excessive effort
- Repetitive tasks
- Exposure to flames
- Extreme weather (hot or cold)
- Other: ______

99. 35.- Type of injury: *

Mark only one oval.

- Burn
- Heatstroke
- Inhalation of fumes
- Muscle pain
- Tendinitis
- Muscle rupture
- Osteo-articular sprain
- Joint dislocation
- Bone fracture
- Fracture
- Wound/cut, with abundant bleeding
- Concussion
- Stroke
- Blisters
- Other: ____________________

100. 36.- Injured body part: *

Mark only one oval.

- Head
- Neck
- Nose
- Eyes
- Ears
- Fingers-hand
- Hand
- Wrist
- Forearm
- Elbow
- Shoulder
- Collar bone
- Upper back (cervical area)
- Middle back (thoracic area)
- Lower back (lumbar area)
- Ribs
- Chest
- Abdomen
- Hip
- Pelvis
- Thigh
- Knee
- Leg
- Ankle
- Foot
- Toenails
- Toes
- Plantar fascia
- Heel
- Other: ____________________

101. 37.-Duration of the lesion: *

Mark only one oval.

- < 1 week
- 1-3 weeks
- 3-6 weeks
- > 6 weeks

102. 38.- The injury required medical attention: *

Mark only one oval.

- No
- Yes

103. 39.- Did the injury require sick leave or absence from work? *

Mark only one oval.

- No
- Yes

Skip to question 104

PART 5: CHRONIC PAIN

We understand chronic pain to be pain of more than 3 months duration that may result from illness, injury, trauma, surgery, or of unknown origin (Levins et al, 2019). In this section you have several questions regarding chronic pain related to the development of your profession.

104. 40- Do you suffer from chronic pain or chronic discomfort related to the development of your profession? *

Mark only one oval.

- No
- Yes

105. 40.1. If yes, indicate where:

Select all that apply.

- [ ] Head
- [ ] Neck
- [ ] Nose
- [ ] Eyes
- [ ] Ears
- [ ] Fingers-hand
- [ ] Hand
- [ ] Wrist
- [ ] Forearm
- [ ] Elbow
- [ ] Shoulder
- [ ] Clavicle
- [ ] Upper back (cervical area)
- [ ] Middle back (thoracic area)
- [ ] Lower back (lumbar area)
- [ ] Ribs
- [ ] Chest
- [ ] Abdomen
- [ ] Hip
- [ ] Pelvis
- [ ] Thigh
- [ ] Knee
- [ ] Leg
- [ ] Ankle
- [ ] Foot
- [ ] Nails - foot
- [ ] Toes foot
- [ ] Plantar fascia
- [ ] Heel
- [ ] Other: ____________

106. 40.2.- What do you think this is due to?

Select all that apply.

- [ ] Poor preparation
- [ ] It's an old injury
- [ ] Training loads (too much training)
- [ ] Training loads (too little training)
- [ ] Bad working postures
- [ ] Poor maintenance of equipment
- [ ] Other: ____________

ACKNOWLEDGMENTS

We greatly appreciate you taking a few minutes of your time to fill out this questionnaire. We would also appreciate it if you could share the questionnaire with fellow BRIF professionals, in order to achieve a representative participation, and to be able to establish valid and objective conclusions.

107. If you are interested in receiving the results of this research, please specify your EMAIL below.

____

108. THANK YOU FOR YOUR TIME. If you have any comments or clarifications you would like to make about the questionnaire please use the following space (with any questions please do not hesitate to contact us: fgarh@unileon.es or patxi.leon@deusto.es)

____

This content has not been created or approved by Google.

Google Forms
