# Supplementary material for: Occupational Injuries of Spanish Wildland Firefighters: A Descriptive Analysis
Source: Healthcare (Basel). 2024 Aug 13;12(16):1615. doi: 10.3390/healthcare12161615 (PMC11354085; doi:10.3390/healthcare12161615)
Supplement: Supplementary file 1 [file healthcare-12-01615-s001.zip › Questionnaire (Spanish).pdf]

ANÁLISIS DESCRIPTIVO DE LAS PRINCIPALES LESIONES DE LAS BRIGADAS DE REFUERZO CONTRA INCENDIOS FORESTALES (BRIF)

A grandes rasgos, la labor de extinción de Incendios Forestales se constata como una labor eminentemente física muy exigente, dónde la larga duración del esfuerzo, condiciones climáticas hostiles y cambiantes, y la larga duración del esfuerzo conlleva al trabajador a una carga física muy elevada. En otras profesiones físicas tipo Bomberos de Estructura y Militares, se han descrito las principales lesiones y el riesgo potencial de sufrirlas en numerosos estudios, pero no es el caso del Bomberos Forestal. Por lo que el presente cuestionario, creado en conjunto por miembros de la Universidad de Deusto-Donosti (patxi.leon@deusto.es), y de la Universidad de León (fgarh@unileon.es), y con el apoyo de la Asociación de Trabajadores BRIF (AT-BRIF), tiene el objetivo de conocer y poder profundizar en la tipología de las lesiones de los Bomberos Forestales BRIF.

El cuestionario consta de 5 partes:
1ª Parte: variables sociodemográficas (personales y laborales)
2ª Parte: actividad física realizada en su vida diaria
3ª Parte: aspectos comportamentales y psicológicos
4ª Parte: lesiones producidas dentro del ámbito laboral
5ª Parte: dolor crónico

La participación es totalmente voluntaria y anónima.

El tratamiento, la comunicación y la cesión de los datos de carácter personal de todos los sujetos participantes se ajustará a lo dispuesto en la Ley Orgánica 3/2018, de 5 de diciembre, de Protección de Datos Personales y garantía de los derechos digitales. De acuerdo a lo que establece la legislación mencionada, usted puede ejercer los derechos de oposición y cancelación de datos, para lo cual deberá dirigirse a los directores del estudio. Los investigadores del proyecto podrán tener acceso a los datos del voluntario. Los datos personales y la información obtenida de este estudio, con garantía de privacidad para su identidad, se conocerá sólo por los investigadores del proyecto. Con la aceptación de este consentimiento usted autoriza la recogida, almacenamiento y análisis de sus datos solicitados, desvinculados de la identidad por un sistema de codificación doble reversible. Aquellas personas que continúen el proceso mostrarán su aceptación a las características de la investigación y asegurará su comprensión.

*Obligatorio

1. 1.- He leído y acepto las condiciones: *

Marca solo un óvalo.

- ◯ Sí
- ◯ No

VARIABLES SOCIODEMOGRÁFICAS

En este apartado se preguntan cuestiones acerca de las características personales y laborales de los participantes, con el fin de contextualizar la muestra de una forma objetiva

2. 2.- Edad: *

3. 3.- Sexo: *

Marca solo un óvalo.

- Mujer
- Hombre
- Otro

4. 4.- Peso en Kg: *

5. 5.- Estatura en cm: *

6. 6.- ¿Eres fumador? *

Marca solo un óvalo.

- No
- Sí

7. 6.1- En el caso de ser fumador. ¿Cuántos cigarrillos fumas un día normal?

Marca solo un óvalo.

- 3 o menos
- De 4 a 10
- De 11 a 15
- 16 o más

8. 7.- ¿ Consumes alcohol? *

Marca solo un óvalo.

- No
- Sí, de forma ocasional (fin de semana)
- Sí, de forma habitual (a diario)

9. 8.- ¿ Has pasado la COVID- 2019? *

Marca solo un óvalo.

- No
- No lo sé con certeza
- Sí, asintomático
- Sí, síntomas leves
- Sí, síntomas graves sin hospitalización
- Sí, síntomas graves con hospitalización

10. 8.1.- En el caso de haber pasado la enfermedad de la COVID- 19, señala cuándo:

Marca solo un óvalo.

- Marzo 2020
- Abril 2020
- Mayo 2020
- Junio 2020
- Julio 2020
- Agosto 2020
- Septiembre 2020
- Octubre 2020
- Noviembre 2020
- Diciembre 2020
- Enero 2021
- Febrero 2021

11. 9.- Años de experiencia dentro del dispositivo BRIF: *

12. 10.- BRIF en la que trabajas actualmente (en el caso de ser destinado este año a Ruente, señala en la que tienes destino habitual + la pestaña de Ruente): *

Selecciona todos los que correspondan.

- [ ] Tineo
- [ ] Laza
- [ ] Lubia
- [ ] Puerto del Pico
- [ ] Tabuyo del monte
- [ ] Daroca
- [ ] Pinofranqueado
- [ ] La Iglesuela
- [ ] Prado de los Esquiladores
- [ ] Puntagorda
- [ ] Ruente

13. 11.- Puesto actual: *

Marca solo un óvalo.

- ( ) Especialista bombero/a forestal
- ( ) Capataz bombero/a forestal
- ( ) Técnico/a bombero/a forestal

14. 12.- Aproximadamente, peso del EPI + complementos utilizados (en kg) (ej: cantimplora, material de avituallamiento, ropa de cambio, etc.,):

____

15. 13.- Principales herramientas utilizadas durante la campaña de extinción: (señala las 2 más utilizadas) *

Selecciona todos los que correspondan.

- [ ] Mochila extintora
- [ ] Batefuegos
- [ ] Mochila extintora + batefuegos
- [ ] Pulaski, azada, gorgui, mcleod o similar
- [ ] Calabozo, tajamatas o similar
- [ ] Motosierra, cargas de la motosierra, etc.
- [ ] Herramienta rotatoria en función de cuadrante
- [ ] Carga de material, alimentos, avituallamiento, etc.
- [ ] Labores de gestión, control y/o organización

Otro: [ ] _______________________

16. 14.- Principales herramientas/labores realizadas durante fase de labores preventivas (invierno): *

Selecciona todos los que correspondan.

- [ ] Desbrozadora
- [ ] Motosierra
- [ ] Otras herramientas mecánicas
- [ ] Labores de gestión, organización y/o control
- [ ] Acarreo de ramas o material

Otro: [ ] ____________________

17. 15.- En el caso de participar en quemas prescritas, y/o EPRIF indica las principales herramientas /acciones utilizadas/realizadas:

Selecciona todos los que correspondan.

- [ ] Antorcha de goteo
- [ ] Mochila extintora
- [ ] Batefuego
- [ ] Desbrozadora
- [ ] Motosierra
- [ ] Acarreo de ramas o material
- [ ] Rotación de funciones/ herramientas según cuadrante
- [ ] Labores de gestión, control y/o organización

Otro: [ ] ____________________

ACTIVIDAD FÍSICA

En este apartado se van a preguntar cuestiones acerca de la actividad física realizada durante tu día a día.

18. 16.- ¿Cuántas horas de media entrenas a la semana? *

Marca solo un óvalo.

- ( ) <1
- ( ) 1-3
- ( ) 3-5
- ( ) 5-7
- ( ) >7

19. 17.- Normalmente, en una semana típica: ¿cuántos días a la semana eres físicamente activo durante al menos 60 minutos? (La actividad física es cualquier actividad que incrementa tu ritmo cardíaco y te hace estar falto de aire en algún momento. Esta actividad física puede ser realizada en cualquier deporte, jugando con amistades, o caminando al trabajo) *

Marca solo un óvalo.

- Ningún día
- Un día
- Dos días
- Tres días
- Cuatro días
- Cinco días
- Seis días
- Siete días

20. 18. -¿Qué tipo de actividad física realizas? *

Selecciona todos los que correspondan.

- Caminata
- Senderismo
- Carrera
- Entrenamieto de fuerza
- Deportes de equipo (ej: rugby, fútbol, etc.)
- Deporte individual (ej: tenis, piragüismo, etc.)

Otro: ____________________

21. 18.1- ¿Realizas ejercicios específicos para el fortalecimiento del CORE? *

Marca solo un óvalo.

- No
- Sí

22. 19.- ¿Piensas que eres una persona activa comparada con otra gente de su edad? *

Marca solo un óvalo.

- Muy inactiva
- Inactiva
- Neutra
- Activa
- Muy activa

23. 20.- ¿Cómo describes tu salud en general? *

Marca solo un óvalo.

- Muy débil
- Débil
- Neutra
- Buena
- Muy buena

24. 20.1- De manera habitual, la calidad de tu sueño fue: *

Marca solo un óvalo.

- Muy mala
- Mala
- Pasable
- Buena
- Muy buena

3ª PARTE: ASPECTOS RELACIONADOS CON EL ESTADO EMOCIONAL

En el siguiente apartado se desarrollan 7 preguntas relacionadas con el estrés y estados de ánimo

25. 21.- En el último mes... ¿Con qué frecuencia te has sentido incapaz de controlar cosas importantes de tu vida? *

Marca solo un óvalo.

- Nunca
- Casi nunca
- De vez en cuando
- Frecuentemente
- Muy a menudo

26. 22.- En el último mes... ¿Con qué frecuencia has estado seguro de tu capacidad para manejar tus problemas? *

Marca solo un óvalo.

- Nunca
- Casi nunca
- De vez en cuando
- Frecuentemente
- Muy a menudo

27. 23.- En el último mes...¿Con qué frecuencia has sentido que las cosas te iban bien? *

Marca solo un óvalo.

- Nunca
- Casi nunca
- De vez en cuando
- Frecuentemente
- Muy a menudo

28. 24.- En el último mes... ¿Con qué frecuencia has sentido que las dificultades se acumulaban tanto que no podías superarlas? *

Marca solo un óvalo.

- Nunca
- Casi nunca
- De vez en cuando
- Frecuentemente
- Muy a menudo

29. 25. Tus pensamientos son (donde calma=0, y preocupaciones=10): *

Marca solo un óvalo.

	0	1	2	3	4	5	6	7	8	9	10	
Calma												Preocupación

30. 26.- Tu cuerpo se siente (donde relajado= 0, y tenso=10): *

Marca solo un óvalo.

	0	1	2	3	4	5	6	7	8	9	10	
Relajado												Tenso

31. 27.- Cómo te sientes (donde seguro= 0, y asustado=10): *

Marca solo un óvalo.

	0	1	2	3	4	5	6	7	8	9	10	
Seguro												Asustado

4ª PARTE: LESIONES OCUPACIONALES

Primeramente y atendiendo a lo descrito por Phelps et al, (2018), "Una lesión se considera relacionada con el trabajo (ocupacional) si un evento o exposición en el ambiente de trabajo causó o contribuyó a la lesión o agravó significativamente una lesión ya existente". Así mismo se entiende que la lesión no implica directamente baja laboral, sino que puede existir o no, pero en todos los casos produce en el trabajador molestias y/o dolor, condicionando la calidad de vida y laboral del mismo (Scherzer et al, 2005).

Dentro del ambiente del trabajo entendemos: incendios o emergencias (desplazamiento, actuación o regreso), entrenamiento físico en base, prácticas técnico-tácticas (marchas, manejo de herramientas, etc), labores preventivas, quemas prescritas, etc.

En este apartado encontrarás distintas preguntas relacionadas con las lesiones producidas en el ámbito laboral.

32. 28.- ¿Has tenido alguna lesión relacionada con el trabajo? *

Marca solo un óvalo.

- No *Salta a la pregunta 104*
- Sí

LESIÓN 1

33. 29.- En el caso de haber sufrido alguna lesión relacionada con el trabajo, ¿Cuándo tuvo lugar la lesión?: *

Marca solo un óvalo.

- Durante el último año (entre febrero 2020 y el momento actual)
- Hace más de un año

34. 30.- Si la lesión fue en el último año, indica el mes en el que ocurrió:

Marca solo un óvalo.

- Febrero 2020
- Marzo 2020
- Abril 2020
- Mayo 2020
- Junio 2020
- Julio 2020
- Agosto 2020
- Septiembre 2020
- Octubre 2020
- Noviembre 2020
- Diciembre 2020
- Enero 2021
- Febrero 2021

35. 31.- Puesto de trabajo que ocupabas cuando se produjo la lesión: *

Marca solo un óvalo.

- Especialista Bombero/a Forestal
- Capataz Bombero/a Forestal
- Técnico/a Bombero/a Forestal

36. 32.- ¿Qué tipo de lesión fue? *

Marca solo un óvalo.

- Nueva lesión
- Recaída

37. 33.-Actividad que estabas realizando cuando ocurrió la lesión: *

Marca solo un óvalo.

- Incendio Forestal
- Otras emergencias
- Prácticas en base
- Entrenamiento físico en base
- Labores preventivas
- Otro: ______________________

38. 33.1.- En el caso de haber sufrido la lesión durante un "Incendio Forestal", señala la labor realizada en el momento de la misma:

Marca solo un óvalo.

- Aproximación
- Ataque directo
- Ataque Indirecto
- Mixto
- Vigilancia
- Labores de perimetración, liquidación
- Retirada
- No recordado
- Otro: ______________________

39. 33.2.-En el caso de haber sufrido la lesión durante "prácticas en base" señala la labor realizada en el momento de la misma:

Marca solo un óvalo.

- Manejo de herramientas (línea de defensa, practica de ataque directo, etc.)
- Marchas con EPI
- Marchas con EPI + herramienta
- Adecuación de material (limpieza, afilado, etc)
- Embarques y desembarques
- No recordado
- Otro: ______________________

40. 33.3.-En el caso de haber sufrido la lesión durante "entrenamiento físico en base" señala el tipo de entrenamiento realizado en el momento de la misma:

Marca solo un óvalo.

- Entrenamiento de fuerza
- Entrenamiento de resistencia
- Entrenamiento de flexibilidad/movilidad
- No recordado
- Otro: ______________________

41. 33.4.-En el caso de haber sufrido la lesión durante "labores preventivas" señala el tipo de tarea o labor realizada en el momento de la misma:

Marca solo un óvalo.

- Desbroce
- Quemas prescritas
- Acceso
- Transporte de cargas
- Acarreo de material y ramas
- Quemas de restos de material
- No recordado
- Otro: ______________________

42. 34.- Causa principal de la lesión: *

Marca solo un óvalo.

- Impacto
- Sobreuso
- Desconocida
- Otro: ______________________

43. 34.1.- En el caso de haber seleccionado la opción IMPACTO, señala la opción que ocasionó la lesión:

Marca solo un óvalo.

- Caída
- Salto
- Resbalón
- Tropiezo
- Golpeo de un objeto
- Otro: ______________________

44. 34.2.- En el caso de haber seleccionado la opción SOBREUSO, señala la opción que ocasionó la lesión:

Marca solo un óvalo.

- Esfuerzo excesivo
- Tareas repetitivas
- Exposición a las llamas
- Clima extremo (frío o calor)
- Otro: ______________________

45. 35.- Tipo de lesión: *

Marca solo un óvalo.

- Quemadura
- Insolación
- Inhalación de humos
- Dolor muscular
- Tendinitis
- Rotura muscular
- Esguince osteo-articular
- Dislocación articular
- Fisura ósea
- Fractura
- Herida/corte, con sangrado abundante
- Conmoción cerebral
- Golpe
- Ampollas
- Otro: ____________________

46. 36.- Parte del cuerpo lesionada: *

Marca solo un óvalo.

- Cabeza
- Cuello
- Nariz
- Ojos
- Oídos
- Dedos- mano
- Mano
- Muñeca
- Antebrazo
- Codo
- Hombro
- Clavícula
- Espalda alta (zona cervical)
- Espalda media (zona torácica)
- Espalda baja (zona lumbar)
- Costillas
- Pecho
- Abdomen
- Cadera
- Pelvis
- Muslo
- Rodilla
- Pierna
- Tobillo
- Pie
- Uñas- pie
- Dedos píe
- Fascia plantar
- Talón
- Otro: ______________________

47. 37.-Duración de la lesión: *

Marca solo un óvalo.

- < 1 semana
- 1-3 semanas
- 3-6 semanas
- > 6 semanas

48. 38.- La lesión requirió atención médica: *

Marca solo un óvalo.

- No
- Sí

49. 39.- ¿La lesión requirió baja o ausencia al puesto de trabajo? *

Marca solo un óvalo.

- No
- Sí

50. ¿Has tenido más lesiones en el trabajo?

Marca solo un óvalo.

- No *Salta a la pregunta 104*
- Sí

LESIÓN 2

51. 29.- En el caso de haber sufrido alguna lesión relacionada con el trabajo, ¿Cuándo tuvo lugar la lesión?: *

Marca solo un óvalo.

- Durante el último año (entre febrero 2020 y el momento actual)
- Hace más de un año

52. 30.- Si la lesión fue en el último año, indica el mes en el que ocurrió:

Marca solo un óvalo.

- Febrero 2020
- Marzo 2020
- Abril 2020
- Mayo 2020
- Junio 2020
- Julio 2020
- Agosto 2020
- Septiembre 2020
- Octubre 2020
- Noviembre 2020
- Diciembre 2020
- Enero 2021
- Febrero 2021

53. 31.- Puesto de trabajo que ocupabas cuando se produjo la lesión: *

Marca solo un óvalo.

- Especialista Bombero/a Forestal
- Capataz Bombero/a Forestal
- Técnico/a Bombero/a Forestal

54. 32.- ¿Qué tipo de lesión fue? *

Marca solo un óvalo.

- Nueva lesión
- Recaída

55. 33.-Actividad que estabas realizando cuando ocurrió la lesión: *

Marca solo un óvalo.

- Incendio Forestal
- Otras emergencias
- Prácticas en base
- Entrenamiento físico en base
- Labores preventivas
- Otro: ______________________

56. 33.1.- En el caso de haber sufrido la lesión durante un "Incendio Forestal", señala la labor realizada en el momento de la misma:

Marca solo un óvalo.

- Aproximación
- Ataque directo
- Ataque Indirecto
- Mixto
- Vigilancia
- Labores de perimetración, liquidación
- Retirada
- No recordado
- Otro: ____________________

57. 33.2.-En el caso de haber sufrido la lesión durante "prácticas en base" señala la labor realizada en el momento de la misma:

Marca solo un óvalo.

- Manejo de herramientas (línea de defensa, practica de ataque directo, etc.)
- Marchas con EPI
- Marchas con EPI + herramienta
- Adecuación de material (limpieza, afilado, etc)
- Embarques y desembarques
- No recordado
- Otro: ____________________

58. 33.3.-En el caso de haber sufrido la lesión durante "entrenamiento físico en base" señala el tipo de entrenamiento realizado en el momento de la misma:

Marca solo un óvalo.

- Entrenamiento de fuerza
- Entrenamiento de resistencia
- Entrenamiento de flexibilidad/movilidad
- No recordado
- Otro: ____________________

59. 33.4.-En el caso de haber sufrido la lesión durante "labores preventivas" señala el tipo de tarea o labor realizada en el momento de la misma:

Marca solo un óvalo.

- Desbroce
- Quemas prescritas
- Acceso
- Transporte de cargas
- Acarreo de material y ramas
- Quemas de restos de material
- No recordado
- Otro: ____________________

60. 34.- Causa principal de la lesión: *

Marca solo un óvalo.

- Impacto
- Sobreuso
- Desconocida
- Otro: ____________________

61. 34.1.- En el caso de haber seleccionado la opción IMPACTO, señala la opción que ocasionó la lesión:

Marca solo un óvalo.

- Caída
- Salto
- Resbalón
- Tropiezo
- Golpeo de un objeto
- Otro: ____________________

62. 34.2.- En el caso de haber seleccionado la opción SOBREUSO, señala la opción que ocasionó la lesión:

Marca solo un óvalo.

- Esfuerzo excesivo
- Tareas repetitivas
- Exposición a las llamas
- Clima extremo (frío o calor)
- Otro: ____________________

63. 35.- Tipo de lesión: *

Marca solo un óvalo.

- Quemadura
- Insolación
- Inhalación de humos
- Dolor muscular
- Tendinitis
- Rotura muscular
- Esguince osteo-articular
- Dislocación articular
- Fisura ósea
- Fractura
- Herida/corte, con sangrado abundante
- Conmoción cerebral
- Golpe
- Ampollas
- Otro: ______________________

64. 36.- Parte del cuerpo lesionada: *

Marca solo un óvalo.

- Cabeza
- Cuello
- Nariz
- Ojos
- Oídos
- Dedos- mano
- Mano
- Muñeca
- Antebrazo
- Codo
- Hombro
- Clavícula
- Espalda alta (zona cervical)
- Espalda media (zona torácica)
- Espalda baja (zona lumbar)
- Costillas
- Pecho
- Abdomen
- Cadera
- Pelvis
- Muslo
- Rodilla
- Pierna
- Tobillo
- Pie
- Uñas- pie
- Dedos píe
- Fascia plantar
- Talón
- Otro: ______________________

65. 37.-Duración de la lesión: *

Marca solo un óvalo.

- < 1 semana
- 1-3 semanas
- 3-6 semanas
- > 6 semanas

66. 38.- ¿La lesión requirió atención médica?

Marca solo un óvalo.

- No
- Sí

67. 39.- ¿La lesión requirió baja o ausencia al puesto de trabajo? *

Marca solo un óvalo.

- No
- Sí

68. ¿Has tenido más lesiones en el trabajo? *

Marca solo un óvalo.

- No *Salta a la pregunta 104*
- Sí

LESIÓN 3

69. 29.- En el caso de haber sufrido alguna lesión relacionada con el trabajo, ¿Cuándo tuvo lugar la lesión?: *

Marca solo un óvalo.

- Durante el último año (entre febrero 2020 y el momento actual)
- Hace más de un año

70. 30.- Si la lesión fue en el último año, indica el mes en el que ocurrió:

Marca solo un óvalo.

- Febrero 2020
- Marzo 2020
- Abril 2020
- Mayo 2020
- Junio 2020
- Julio 2020
- Agosto 2020
- Septiembre 2020
- Octubre 2020
- Noviembre 2020
- Diciembre 2020
- Enero 2021
- Febrero 2021

71. 31.- Puesto de trabajo que ocupabas cuando se produjo la lesión: *

Marca solo un óvalo.

- Especialista Bombero/a Forestal
- Capataz Bombero/a Forestal
- Técnico/a Bombero/a Forestal

72. 32.- ¿Qué tipo de lesión fue? *

Marca solo un óvalo.

- Nueva lesión
- Recaída

73. 33.-Actividad que estabas realizando cuando ocurrió la lesión: *

Marca solo un óvalo.

- Incendio Forestal
- Otras emergencias
- Prácticas en base
- Entrenamiento físico en base
- Labores preventivas
- Otro: ____________________

74. 33.1.- En el caso de haber sufrido la lesión durante un "Incendio Forestal", señala la labor realizada en el momento de la misma:

Marca solo un óvalo.

- Aproximación
- Ataque directo
- Ataque Indirecto
- Mixto
- Vigilancia
- Labores de perimetración, liquidación
- Retirada
- No recordado
- Otro: ______________________

75. 33.2.-En el caso de haber sufrido la lesión durante "prácticas en base" señala la labor realizada en el momento de la misma:

Marca solo un óvalo.

- Manejo de herramientas (línea de defensa, practica de ataque directo, etc.)
- Marchas con EPI
- Marchas con EPI + herramienta
- Adecuación de material (limpieza, afilado, etc)
- Embarques y desembarques
- No recordado
- Otro: ______________________

76. 33.3.-En el caso de haber sufrido la lesión durante "entrenamiento físico en base" señala el tipo de entrenamiento realizado en el momento de la misma:

Marca solo un óvalo.

- Entrenamiento de fuerza
- Entrenamiento de resistencia
- Entrenamiento de flexibilidad/movilidad
- No recordado
- Otro: ______________________

77. 33.4.-En el caso de haber sufrido la lesión durante "labores preventivas" señala el tipo de tarea o labor realizada en el momento de la misma:

Marca solo un óvalo.

- Desbroce
- Quemas prescritas
- Acceso
- Transporte de cargas
- Acarreo de material y ramas
- Quemas de restos de material
- No recordado
- Otro: __________

78. 34.- Causa principal de la lesión: *

Marca solo un óvalo.

- Impacto
- Sobreuso
- Desconocida
- Otro: __________

79. 34.1.- En el caso de haber seleccionado la opción IMPACTO, señala la opción que ocasionó la lesión:

Marca solo un óvalo.

- Caída
- Salto
- Resbalón
- Tropiezo
- Golpeo de un objeto
- Otro: __________

80. 34.2.- En el caso de haber seleccionado la opción SOBREUSO, señala la opción que ocasionó la lesión:

Marca solo un óvalo.

- Esfuerzo excesivo
- Tareas repetitivas
- Exposición a las llamas
- Clima extremo (frío o calor)
- Otro: __________

81. 35.- Tipo de lesión:

Marca solo un óvalo.

- Quemadura
- Insolación
- Inhalación de humos
- Dolor muscular
- Tendinitis
- Rotura muscular
- Esguince osteo-articular
- Dislocación articular
- Fisura ósea
- Fractura
- Herida/corte, con sangrado abundante
- Conmoción cerebral
- Golpe
- Ampollas
- Otro: ______________________

82. 36.- Parte del cuerpo lesionada: *

Marca solo un óvalo.

- Cabeza
- Cuello
- Nariz
- Ojos
- Oídos
- Dedos- mano
- Mano
- Muñeca
- Antebrazo
- Codo
- Hombro
- Clavícula
- Espalda alta (zona cervical)
- Espalda media (zona torácica)
- Espalda baja (zona lumbar)
- Costillas
- Pecho
- Abdomen
- Cadera
- Pelvis
- Muslo
- Rodilla
- Pierna
- Tobillo
- Pie
- Uñas- pie
- Dedos píe
- Fascia plantar
- Talón
- Otro: ______________________

83. 37.-Duración de la lesión: *

Marca solo un óvalo.

- < 1 semana
- 1-3 semanas
- 3-6 semanas
- > 6 semanas

84. 38.- La lesión requirió atención médica

Marca solo un óvalo.

- No
- Sí

85. 39.- ¿La lesión requirió baja o ausencia al puesto de trabajo? *

Marca solo un óvalo.

- No
- Sí

86. ¿Has tenido más lesiones en el trabajo? *

Marca solo un óvalo.

- No *Salta a la pregunta 104*
- Sí

LESIÓN 4

87. 29.- En el caso de haber sufrido alguna lesión relacionada con el trabajo, ¿Cuándo tuvo lugar la lesión?: *

Marca solo un óvalo.

- Durante el último año (entre febrero 2020 y el momento actual)
- Hace más de un año

88. 30.- Si la lesión fue en el último año, indica el mes en el que ocurrió:

Marca solo un óvalo.

- Febrero 2020
- Marzo 2020
- Abril 2020
- Mayo 2020
- Junio 2020
- Julio 2020
- Agosto 2020
- Septiembre 2020
- Octubre 2020
- Noviembre 2020
- Diciembre 2020
- Enero 2021
- Febrero 2021

89. 31.- Puesto de trabajo que ocupabas cuando se produjo la lesión: *

Marca solo un óvalo.

- Especialista Bombero/a Forestal
- Capataz Bombero/a Forestal
- Técnico/a Bombero/a Forestal

90. 32.- ¿Qué tipo de lesión fue? *

Marca solo un óvalo.

- Nueva lesión
- Recaída

91. 33.-Actividad que estabas realizando cuando ocurrió la lesión: *

Marca solo un óvalo.

- Incendio Forestal
- Otras emergencias
- Prácticas en base
- Entrenamiento físico en base
- Labores preventivas
- Otro: ____________________

92. 33.1.- En el caso de haber sufrido la lesión durante un "Incendio Forestal", señala la labor realizada en el momento de la misma:

Marca solo un óvalo.

- Aproximación
- Ataque directo
- Ataque Indirecto
- Mixto
- Vigilancia
- Labores de perimetración, liquidación
- Retirada
- No recordado
- Otro: ______________________

93. 33.2.-En el caso de haber sufrido la lesión durante "prácticas en base" señala la labor realizada en el momento de la misma:

Marca solo un óvalo.

- Manejo de herramientas (línea de defensa, practica de ataque directo, etc.)
- Marchas con EPI
- Marchas con EPI + herramienta
- Adecuación de material (limpieza, afilado, etc)
- Embarques y desembarques
- No recordado
- Otro: ______________________

94. 33.3.-En el caso de haber sufrido la lesión durante "entrenamiento físico en base" señala el tipo de entrenamiento realizado en el momento de la misma:

Marca solo un óvalo.

- Entrenamiento de fuerza
- Entrenamiento de resistencia
- Entrenamiento de flexibilidad/movilidad
- No recordado
- Otro: ______________________

95. 33.4.-En el caso de haber sufrido la lesión durante "labores preventivas" señala el tipo de tarea o labor realizada en el momento de la misma:

Marca solo un óvalo.

- Desbroce
- Quemas prescritas
- Acceso
- Transporte de cargas
- Acarreo de material y ramas
- Quemas de restos de material
- No recordado
- Otro: ____________

96. 34.- Causa principal de la lesión: *

Marca solo un óvalo.

- Impacto
- Sobreuso
- Desconocida
- Otro: ____________

97. 34.1.- En el caso de haber seleccionado la opción IMPACTO, señala la opción que ocasionó la lesión:

Marca solo un óvalo.

- Caída
- Salto
- Resbalón
- Tropiezo
- Golpeo de un objeto
- Otro: ____________

98. 34.2.- En el caso de haber seleccionado la opción SOBREUSO, señala la opción que ocasionó la lesión:

Marca solo un óvalo.

- Esfuerzo excesivo
- Tareas repetitivas
- Exposición a las llamas
- Clima extremo (frío o calor)
- Otro: ____________

99. 35.- Tipo de lesión: *

Marca solo un óvalo.

- Quemadura
- Insolación
- Inhalación de humos
- Dolor muscular
- Tendinitis
- Rotura muscular
- Esguince osteo-articular
- Dislocación articular
- Fisura ósea
- Fractura
- Herida/corte, con sangrado abundante
- Conmoción cerebral
- Golpe
- Ampollas
- Otro: ____________________

100. 36.- Parte del cuerpo lesionada: *

Marca solo un óvalo.

- Cabeza
- Cuello
- Nariz
- Ojos
- Oídos
- Dedos- mano
- Mano
- Muñeca
- Antebrazo
- Codo
- Hombro
- Clavícula
- Espalda alta (zona cervical)
- Espalda media (zona torácica)
- Espalda baja (zona lumbar)
- Costillas
- Pecho
- Abdomen
- Cadera
- Pelvis
- Muslo
- Rodilla
- Pierna
- Tobillo
- Pie
- Uñas- pie
- Dedos píe
- Fascia plantar
- Talón
- Otro: ______________________

101. 37.-Duración de la lesión: *

Marca solo un óvalo.

- < 1 semana
- 1-3 semanas
- 3-6 semanas
- > 6 semanas

102. 38.- La lesión requirió atención médica: *

Marca solo un óvalo.

- No
- Sí

103. 39.- ¿La lesión requirió baja o ausencia al puesto de trabajo? *

Marca solo un óvalo.

- No
- Sí

Salta a la pregunta 104

5ª PARTE: DOLOR CRÓNICO

Entendemos que el dolor crónico es un dolor de más de 3 meses de duración que puede resultar de una enfermedad, lesión, traumatismo, cirugía o de origen desconocido (Levins et al, 2019). En este apartado dispones de varias preguntas en relación al dolor crónico relacionado con el desarrollo de tu profesión.

104. 40- ¿Sufres dolor crónico o molestias crónicas relacionadas con el desarrollo de tu profesión? *

Marca solo un óvalo.

- No
- Sí

105. 40.1. Si has respondido si, indica donde:

Selecciona todos los que correspondan.

- [ ] Cabeza
- [ ] Cuello
- [ ] Nariz
- [ ] Ojos
- [ ] Oídos
- [ ] Dedos- mano
- [ ] Mano
- [ ] Muñeca
- [ ] Antebrazo
- [ ] Codo
- [ ] Hombro
- [ ] Clavícula
- [ ] Espalda alta (zona cervical)
- [ ] Espalda media (zona torácica)
- [ ] Espalda baja (zona lumbar)
- [ ] Costillas
- [ ] Pecho
- [ ] Abdomen
- [ ] Cadera
- [ ] Pelvis
- [ ] Muslo
- [ ] Rodilla
- [ ] Pierna
- [ ] Tobillo
- [ ] Pie
- [ ] Uñas- pie
- [ ] Dedos píe
- [ ] Fascia plantar
- [ ] Talón

Otro: [ ] ____________________

106. 40.2.- ¿A qué crees que se debe?

Selecciona todos los que correspondan.

- [ ] Mala preparación
- [ ] Es una vieja lesión
- [ ] Cargas de entrenamiento (demasiado entrenamiento)
- [ ] Cargas de entrenamiento (poco entrenamiento)
- [ ] Malas posturas laborales
- [ ] Mantenimiento deficiente del material

Otro: [ ] ____________________

AGRADECIMIENTOS

Agradecemos enormemente que hayas dedicado unos minutos de tu tiempo en rellenar el presente cuestionario. Del mismo modo agradeceríamos que compartas el cuestionario con compañeros de profesión BRIF, con el fin de conseguir una participación representativa, y poder establecer conclusiones válidas y objetivas.

107. Si estás interesado en recibir los resultados de esta investigación especifica tu CORREO ELECTRÓNICO a continuación

108. GRACIAS POR TU TIEMPO. Si tienes algún comentario o aclaración que desees realizar sobre el cuestionario utilice el siguiente espacio (con cualquier consulta no dude en contactarnos: fgarh@unileon.es o patxi.leon@deusto.es)

Este contenido no ha sido creado ni aprobado por Google.

Google Formularios
